# Retraction Note: Molecular and microscopic characterization of a novel Eastern grey kangaroopox virus genome directly from a clinical sample

**DOI:** 10.1038/s41598-024-69649-0

**Published:** 2024-08-19

**Authors:** Subir Sarker, Hayley K. Roberts, Naomie Tidd, Shayne Ault, Georgia Ladmore, Andrew Peters, Jade K. Forwood, Karla Helbig, Shane R. Raidal

**Affiliations:** 1 Department of Physiology, Anatomy and Microbiology, School of Life Sciences, La Trobe University, Melbourne, VIC 3086 Australia; 2 Veterinary Diagnostic Laboratory, Charles Sturt University, Wagga Wagga, NSW 2678 Australia; 3 School of Animal and Veterinary Sciences, Charles Sturt University, Wagga Wagga, NSW 2678 Australia; 4 Lake Road Veterinary Clinic, 327 Lake Albert Road, Kooringal, NSW 2650 Australia; 5 School of Biomedical Sciences, Charles Sturt University, Wagga Wagga, NSW 2678 Australia

Retraction of: *Scientific Reports* 10.1038/s41598-017-16775-7, published online 28 November 2017

The Editors have retracted this Article.

After the publication of this study, concerns were raised about the differences between the genome sequences of the New South Wales and Sunshine Coast isolates of the Easter grey kangaroopox^1^. Tu and Upton demonstrate that these discrepancies are most likely a result of errors in the assembly of the sequencing data reported in this Article. The Editors therefore no longer have confidence in the results and conclusions presented.

Andrew Peters agrees with retraction and its wording. Subir Sarker disagrees with this retraction. Naomie Tidd, Jade K. Forwood, and Karla Helbig have not responded to correspondence from the Editors about this retraction. The Editors were not able to establish the current contact details for Hayley K. Roberts, Shayne Ault, Georgia Ladmore and Shane R. Raidal.
